# Evaluating a computer-based body exposure paradigm for the treatment of body image disturbance in adolescent Anorexia Nervosa: effects on the attentional bias and emotions

**DOI:** 10.3389/fpsyg.2024.1483623

**Published:** 2024-12-16

**Authors:** Lena Sasse, Valeska Stonawski, Oliver Kratz, Gunther Moll, Stefanie Horndasch

**Affiliations:** Department for Child and Adolescent Mental Health, University Hospital Erlangen, Friedrich-Alexander University Erlangen-Nürnberg (FAU), Erlangen, Germany

**Keywords:** Anorexia Nervosa, body image disturbance, body exposure, eyetracking, attentional bias, adolescence

## Abstract

**Background:**

A diagnostic criterion of Anorexia Nervosa (AN) is body image disturbance. Body exposure therapy is a widely used approach to treat this; however, it is unclear which part of body exposure therapy is relevant for regaining a realistic perspective on the own body. This study aimed to examine the role of the attentional bias (AB), which AN patients exhibit to the most disliked parts of their body. Additionally, emotional responses to the body exposure sessions were examined.

**Methods:**

Participants were adolescent girls with a diagnosis of AN, who were randomly assigned to either an intervention (INT) or a treatment-as-usual (TAU) group. Both groups completed a pre and a post session, which included the completion of questionnaires to measure AN-psychopathology. The INT group received four sessions of a computer-based body exposure between the pre and the post session. The viewing pattern was recorded before and after each intervention session via an eyetracking (ET) device, as were emotional response scores on a visual analog scale (VAS; anxiety and disgust). The TAU group did not receive the intervention, but viewing patterns were recorded during the pre and the post session. All participants were asked to list their three least favorite body parts to be able to subsequently assess the AB.

**Results:**

Fifty-eight adolescent girls with AN participated in the study. There were no differences in psychopathology pre to post session, as measured by administered questionnaires. The existence of an AB could be replicated, but there was no significant reduction in the AB pre to post session in the INT group, nor was there an interaction between group and time. Also, no changes in the AB were found within and between sessions in the INT group. Anxiety scores reduced significantly across sessions while disgust scores were significantly higher post session than they were pre session.

**Conclusions:**

While the existence of an AB was demonstrated, the carried-out body exposures were neither sufficient to reduce the AB nor the psychopathology; nevertheless, a significant decrease in anxiety levels showed the usefulness of the exposure sessions. Future research might benefit from more exposure sessions and incorporating AB modification training (ABMT).

## 1 Introduction

Anorexia Nervosa (AN) is a prevalent psychiatric disorder among adolescents, the majority of whom are girls with a peak age of onset at ~15 years of age (Bühren et al., 2017; Solmi et al., 2022). AN has a poor treatment prognosis, resulting in it being the mental illness with the highest mortality rate (Smink et al., 2013). According to ICD-11 criteria, AN is characterized by a significant loss of weight, leading to a body mass index (BMI) that is at least < 18.5 kg^2^/m^2^ for adults or < 5th BMI age percentile for children and adolescents, respectively [World Health Organization (WHO, 2019/2021)]. Furthermore, individuals experience an extreme fear of weight gain, body image disturbance and high body dissatisfaction, which shows the high relevance of shape and weight in AN (Cash and Deagle, 1997; Fairburn and Harrison, 2003). In line with this, patients with AN show a particularly strong reaction to being confronted with their own bodies in front of a mirror, i.e., they show a strong increase in negative emotions as well as in negative cognitions (Vocks et al., 2007). This negative arousal upon being confronted with the own body can lead to both body avoidance and body checking behaviors (Glashouwer et al., 2019). While the former shows in behaviors such as avoiding mirrors, scales and situations in which oneself or others would see one's body (Rosen et al., 1991), the latter may include repeatedly measuring body parts and weighing oneself, as well as critically examining one's shape in the mirror. Body avoidance and body checking can be interpreted as being the behavioral implementation of negative body image (Glashouwer et al., 2019).

Another mitigating role in perpetuating negative body image in AN seems to be an attentional bias (AB), i.e., a skewed perception of the body, which occurs due to dysfunctional cognitive schemata (Aspen et al., 2013). A possible explanation for particularly strong ABs in patients with AN is the assumption of these patients having a highly developed self-schema based on body size and shape cues (Bauer et al., 2017). Hence, the self-schema distorts information processing in a way that results in dysfunctional visual scanning behaviors, which then perpetuates negative body image, as mentioned above. Corresponding to this, Kerr-Gaffney et al. (2018) state that individuals with AN have an AB for body parts they categorize as being unattractive and that the more one dislikes one's/their body, the stronger this AB is. Furthermore, Toh et al. (2020) found that AN patients have a particular AB to body parts which display information about weight status (i.e., lower and mid-torso regions, including the waist, stomach, buttocks, hips and thighs; see also Horndasch et al., 2012). Put together, the combination of high body dissatisfaction, a bias toward looking at body zones that display information about weight status, as well as having an AB toward body parts that are judged as being most unattractive, may lead to a vicious cycle that patients are unable to get out of.

Furthermore, viewing their own body (body exposure) evokes negative emotions, such as anxiety and disgust, in patients with AN. In this context, findings from Melles and Jansen (2023) support the assumed usefulness of exposures to reduce anorexic fears. More generally, exposure therapy has been described as the “gold standard” for the treatment of anxiety disorders (Van Loenen et al., 2022). It aims to reduce fear responses by altering pathological fear structures through activating the respective structure and subsequently providing information that is incongruent with it (Foa and McLean, 2016). It is important for the fear structure to be activated, as it cannot be modified otherwise. The same principle can be applied to body exposures, i.e., the body is the fear-inducing stimulus, in relation to which the fear structure needs to be modified. Interestingly, state anxiety also seems to be a stable predictor for the strength of the AB (Radix et al., 2023). While specific phobias have been shown to be treatable even in a single exposure session (Öst, 1989), more complex, fear-related disorders need several exposure sessions to modify existing fear structures [e.g., 10-12 exposure sessions for posttraumatic stress disorder (Sripada and Rauch, 2015) or 9–10 exposure sessions for social anxiety disorder (Jeong et al., 2021)]. Additionally, disgust might play a particularly relevant role in the maintenance of AN, as proposed by Glashouwer and de Jong (2021). Their proposition stems from the idea that individuals with AN internalize moral ideas (such as a societal notion of having to be thin) to a very high degree, so that when these are incongruent with their own feelings (e.g. “feeling fat”), a (self-) disgust response is evoked (Glashouwer and de Jong, 2021). This often results in body image avoidance, as individuals with AN try to avoid feelings of disgust (Espeset et al., 2012).

As Vocks et al. (2018) report, there have been multiple findings that body exposure therapy yields positive results on behavior associated with body image disturbance. Indeed, body exposure sessions are part of many structured programmes for the treatment of body image disturbance in AN, which have been developed and tested (Herpertz et al., 2018). Nonetheless, it should not be neglected that studies, such as one by Böse (2002), could not find significant improvements in all areas related to body image, despite finding generally positive effects of a body image group therapy. Tuschen-Caffier et al. (2015) state that—because individuals with AN focus more on negatively evaluated parts of their bodies, which perpetuates negative body image—attention should be directed toward neutrally and/or positively evaluated body parts, which are otherwise neglected. However, it needs to be highlighted that the number of studies on adolescent AN is scarce, while there are more studies on such programmes for adult AN. Nevertheless, there are a few studies on adolescent AN, such as one by Biney et al. (2021) who tested “practical body image” (PBI) therapy for children and adolescents with AN. PBI therapy consists of 14 sessions of talking about body perception, addressing challenges therein, as well as six sessions of mirror exposure. For our study, we used a modified version of the body exposure guidance from the manual “*Body image therapy in Anorexia and Bulimia Nervosa: a cognitive-behavioral treatment program”* by Vocks et al. (2018) to carry out the body exposure with our participants. Vocks et al. (2018) evaluated their own manual for body image therapy in several pilot studies, showing significant improvements in participants' body dissatisfaction, body checking behavior, a reduction of the intensity of negative cognitions in relation to their own bodies and an overall reduction of ED symptomatology (Legenbauer et al., 2011). Intrasession effects were analyzed in another evaluation (Vocks et al., 2009), using the body image states scale (Cash et al., 2002), which showed a decrease of body dissatisfaction within session. Hence, the authors of the manual (Vocks et al., 2018) came to the conclusion that their manual is a suitable instrument to treat body image disturbance in individuals with EDs. Nevertheless, they acknowledge that it is unclear which part of their intervention is the crucial one, i.e., the one responsible for improvements in participants' body image disturbance. Hence, it is unclear what role the body exposure sessions, and particularly the AB, play in the treatment of body image disturbance in adolescent AN. Our study aimed to move away from traditional mirror exposures, instead employing a photo-based approach. On one hand, this may ease the process of looking at one's body in a gentler way. On the other hand, it might have the advantage that looking at photos rather than a mirror is more similar to how adolescents see bodies on social media, hence aligning it to a growing part of virtual adolescent life. To distinguish the possible effects of the intervention from the regular treatment effects, we also employed a treatment-as-usual group (TAU).

Our hypotheses for this study were:

1) At the post session, AN-psychopathology, in particular body dissatisfaction, body image avoidance, and body checking, will have decreased more in the INT than in the TAU group.
2) The AB score will be reduced after the intervention in the INT group, but not in the TAU group.
3) The AB will decrease within (pre to post session) and between sessions (sessions one to four) in the INT group.
4) We will investigate the relationship between the AB and AN-psychopathology: We hypothesize that a stronger AB correlates with higher body dissatisfaction, body image avoidance, as well as body checking behaviors.
5) Anxiety and disgust levels will decrease within and between sessions.

## 2 Methods

A study protocol has previously been published; please see Stonawski et al. (2022) for more detailed information on the methods. Not all parameters from the original study were taken into account for the analyses in this paper. Ethical approval for the study was granted by the medical faculty of Friedrich-Alexander-University Erlangen, Germany. The approval is in line with the declaration of Helsinki.

### 2.1 Participants

Recruited were female participants between the ages of 10 and 18 years who were in treatment at the University Hospital Erlangen (inpatient or day-clinic treatment). Participants had to have a diagnosis of either typical (F50.00 or F50.01) or atypical (F50.1) AN, according to ICD-10 criteria (Graubner, 2013). Most participants who had atypical AN did not meet diagnostic criteria for typical AN due to an insufficient degree of weight loss and/or having no amenorrhoea. When starting the study, participants had to have reached a BMI-age-percentile of ≥10. Exclusion criteria were the following: substance use, acute state of psychosis, taking sedatives, learning disabilities, insufficient knowledge of the German language, and chronic somatic diseases. All participants and their parents or legal guardians had to sign informed consent.

### 2.2 Materials

An adapted version for adolescents of the body exposure guidance from Vocks et al. (2018) was recorded as an audio file and used to guide participants through the exposures. The audio file was divided into two parts, the first one dealing with head to hands (duration: 10 min), the second one dealing with the torso to legs (duration: 12 min). Participants were asked to describe the respective body parts in a neutral manner using prompts from the audio, such as “What do your eyebrows look like?” and “Are there differences between the left and the right part of your body?” and “What does your upper and your lower belly look like?”. The viewing pattern was recorded by having participants look at previously taken pictures of themselves in frontal and lateral view for 30 s each on a computer screen. The recording was carried out by Eyegaze Analysis System^TM^ (Interactive Minds, Dresden, Germany), which is an infrared video-based binocular eyetracking system with 60 Hz temporal resolution and gaze position accuracy with 0.45° average error. Several questionnaires, including the Eating Disorder Inventory-2 subscales “body dissatisfaction” and “drive for thinness” (EDI-2; Garner and Olmsted, 1991), the Body Image Avoidance Questionnaire (BIAQ; Rosen et al., 1991), and the body checking questionnaire (BCQ; Reas et al., 2002), were used to assess AN-related psychopathology, whereby higher scores indicated stronger psychopathology. The German version of EDI-2 has been shown to have good reliability (internal consistency between α = 0.73 and α = 0.93) and validity scores; see Paul and Thiel (2005). A German version of the BIAQ was validated by Legenbauer et al. (2007) and a German version of the BCQ was validated by Vocks et al. (2008). Hence, these questionnaires have been shown to be valid and reliable instruments. Additionally, participants were asked to rate the subjective attractiveness of different parts of their bodies (hair, face, neck, cleavage, upper arms, chest, back, waist, lower arms, stomach, buttocks, hips, hands, thighs, knees, calves, feet; taken from Vocks et al., 2018) on a seven-point Likert scale (ranging from very negative to very positive) and to list their three least favorite body parts. This was done twice, once prior to and once after the exposure sessions. Participants were also asked to rate their level of anxiety and disgust before and after each intervention session using a visual analog scale (VAS; Hayes and Patterson, 1921) ranging from 0 to 10.

### 2.3 Procedure

**Figure 1:**
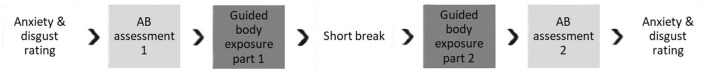
Procedure of a body exposure session. Procedure of a body exposure session for the INT-group.

After giving consent to participate in the study, participants were randomized into an intervention (INT) and a control group (TAU), respectively. To do this, simple randomization was employed, using an online random number generator to generate the allocation sequence. There were no stratification factors. The INT group received six sessions in total, the control group only received two sessions (the first and the last). The first session was a preparatory one, which included taking pictures in short sports clothing (one from the front, one from the side) and filling in questionnaires (see “Materials”). The TAU group was asked to additionally record the viewing pattern. The next four sessions were the body exposure sessions, which only the INT group received and which were carried out in the following order: (1) Rating anxiety and disgust levels on a VAS. (2) Recording the viewing pattern (free viewing time: 30 s in frontal view, immediately followed by 30 s in lateral view). (3) Carrying out the first part of the body exposure, starting from the head and ending at the wrists. (4) Short break to collect other study-relevant data, which is not relevant for this paper. (5) Carrying out the second part of the exposure, starting at the hands and ending with the feet. (6) Recording the viewing pattern again. (7) Rating anxiety and disgust levels again (see also Figure 1).

During the post session, the INT group only filled in the abovementioned questionnaires again while the TAU group was asked to additionally record the viewing pattern again. As the exposure sessions were averagely carried out within the span of two and a half weeks, this duration was matched for the TAU group (i.e., the post session was carried out ~2.5 weeks after the pre session). The TAU group did not carry out body exposures during this time, but they had the opportunity to complete the exposure sessions once they had completed the post session. However, due to the inpatient/day clinic setting, all participants (INT and TAU) were potentially subject to body image content during individual and group therapy sessions. All participants received treatment according to S3-consensus guidelines, including cognitive-behavioral individual and group therapy, as well as nutrition counseling and parents' sessions. The body exposures were performed in addition to the usual treatment methods.

Some information was taken from participants' hospital files, these included: date of birth, height and weight at admission, as well as on the dates of the pre and post sessions (BMI and BMI age percentiles were calculated with this information, along with weight gain from admission to pre session); duration of stay at the hospital until pre session; IQ score (if available); psychiatric diagnoses.

### 2.4 Analysis

#### 2.4.1 Pre-processing of the eyetracking data

ET files were checked for quality of the recorded viewing patterns by multiple researchers, which led to the removal of some data points (due to shifts in the data, lack of recordings, or poor quality of recorded data). The aforementioned shifts in the data occurred in a few instances due to technical errors. To approach this issue, three independent raters identified the degree of data shift; subsequently, files with high inter-rater concordance were shifted back to their original place, while the others were discarded. In order to analyze viewing patterns, all seventeen body parts (see “Materials”) were marked on participants' pictures. Subsequently, time spent looking at the three least favorite body parts (regions of interest; ROIs; these were identified by participants at the pre-session) was calculated against time spent looking at the other body parts in relation to region size to create an AB score:


$$\begin{array}{l} AB\;Score=\;\frac{\frac{\;Time_{\;ROIs}}{Size_{\;ROIs}}}{\frac{Time_{other\;body\;parts}}{Size_{other\;body\;parts}}} \end{array}$$

This was done for each recorded viewing pattern, so there were two scores (frontal and lateral) for each of the eight recorded viewing patterns of the INT group and for each of the two recorded viewing patterns of the TAU group. An AB Score > 1 showed that the participant looked more at their three ROIs than they looked at the rest of their body, i.e., they exhibit an AB toward their least favorite body parts. Hence, the higher the AB Score is, the stronger the AB is. Vice versa, if the AB Score was < 1, this meant the participant did not exhibit an AB. Outliers > ± two SD were removed from the dataset to retain data quality.

#### 2.4.2 Statistical analyses

IBM SPSS Version 28.0.0.0 was used for all statistical analyses. Independent *t*-tests were calculated to check for differences in age, weight gain (from admission to start of the intervention), weeks spent in hospital (from admission to start of the intervention), BMI at pre and post session, IQ (not available for all participants) and number of comorbid psychiatric diagnoses between the INT and TAU group, using Cohen's *d* as a measure of effect size (Cohen, 1988). Subsequently, a two (between-factor: group: INT vs. TAU group) × two (within-factor: time: pre vs. post intervention) mixed ANOVA was employed to analyze potential changes in AN-psychopathology (hypothesis one), as measured by the administered questionnaires EDI (subscales “body dissatisfaction” and “drive for thinness”), BIAQ, and BCQ. After processing the raw ET data according to the abovementioned procedure, linear mixed model analyses were performed. The first LMM analysis was performed to determine the effect of the intervention between the INT and the TAU group, using the AB scores (hypothesis two). To do this, group (INT/TAU), time point (pre/post intervention), as well as the interaction between these were used as fixed effects. Additionally, the different questionnaire scores (EDI subscales, BIAQ, BCQ) were added to the fixed effects as covariates. This also showed whether or not there were differences between the groups. The individual intercept of the participants was used as the random effect. The dependent variable consisted of the AB score in frontal or lateral view, respectively. To explore potential changes between and within intervention sessions (hypothesis three), using only the INT group data, number of viewing pattern (pre/post session), session number (1–4), as well as the interaction between these, were used as fixed effects. Additionally, the different questionnaire scores (EDI subscales, BIAQ, BCQ) were added to the fixed effects as covariates. As before, the individual intercept of the participants was used as the random effect. Also as before, the dependent variable consisted of the AB score in frontal or lateral view, respectively. To check for a link between the strength of the AB and the questionnaire scores, i.e., AN-psychopathology, pre intervention AB scores were correlated with pre intervention questionnaire scores (hypothesis four). Furthermore, another LMM analysis was performed on the anxiety and disgust scores, respectively; using session and pre/post session as fixed factors and participants' individual intercept as the random factor (hypothesis five), using Bonferroni-corrected pairwise comparisons as *post-hoc* tests.

## 3 Results

### 3.1 Participants

Participants were 58 adolescent girls with AN, with a BMI of 16.0–21.5 kg/m^2^ at the start of the intervention (BMI age percentile 7–70), *M* = 17.99 *SD* = 1.08 (BMI age percentile *Mdn* = 16). One participant had a lower BMI age percentile (7) than was officially allowed, which might be due to writing down the daily weight rather than a weekly average at the pre session. Au contraire, the higher end comes about because we also included participants with a diagnosis of atypical AN (*n* = 9). Furthermore, thirty participants had restrictive-type AN while the other nineteen participants had binge/purge type AN. Participants also had a range of comorbid disorders, including depression, generalized anxiety disorder, obsessive compulsive disorder and others (see Table 1). The groups did not differ in age, weight, height, BMI, BMI age percentile, IQ or other measures (see Table 1).

**Table 1:** Participants' demographics.

		**INT (*n* = 33)**	**TAU (*n* = 25)**	**Group comparison INT vs. TAU**		
**Demographics**				***t***	***p***	***d***
Age (in years)	*M (SD)*	14.97 (1.42)	14.92 (1.25)	0.141	0.886	0.04
Weight gain (admission to pre session, in kg)	*M (SD)*	7.68 (3.94)	5.77 (5.19)	1.631	0.108	0.43
Weeks admission to pre session	*M (SD)*	15.05 (7.99)	15.80 (10.06)	−0.318	0.751	0.08
BMI at pre session (in kg/m^2^)	*M (SD)*	18.00 (1.15)	17.98 (0.99)	0.067	0.947	0.02
BMI at post session (in kg/m^2^)	*M (SD)*	18.41 (1.11)	18.23 (0.98)	0.621	0.537	0.17
IQ [*n (INT)* = 24; *n (TAU)* = 18]	*M (SD)*	111.29 (13.30)	112.94 (9.78)	−0.444	0.659	−0.14
**Comorbid psychiatric diagnoses**						
No comorbid diagnosis	*n* (%)	8 (22.86%)	2 (8.33%)			
1 comorbid diagnosis	*n* (%)	18 (51.43%)	14 (58.33%)			
2 comorbid diagnoses	*n* (%)	7 (20.00%)	7 (29.17%)			
>2 comorbid diagnoses	*n* (%)	2 (5.71%)	1 (4.17%)			

Participants' demographics by group (INT, intervention group; TAU, treatment-as-usual group) with mean (M) and standard deviation (SD).

### 3.2 Psychopathology

A 2 (time: pre vs. post intervention) × 2 (group: INT vs. TAU) ANOVA was conducted for each of the questionnaires: EDI (subscales body dissatisfaction, drive for thinness), BIAQ and BCQ. No significant main effects for group or time, nor any interactions between them were found. Nonetheless, there was a trend for the BIAQ: between factor group with *F(1, 53)* = 3.62 and *p* = 0.063 and η^2^ = 0.064 (higher scores in the TAU group) and the within factor time with *F*_(1, 53)_ = 3.17 and *p* = 0.081 and η^2^ = 0.056 (lower scores at the post session). However, there was no interaction effect: *F*_(1, 53)_ = 0.35 and *p* = 0.558 and η^2^ = 0.007. See Table A1 for full table of results and Table 2 for an overview of participants' scores pre and post intervention.

**Table 2:** Questionnaire scores (overview).

		**Pre intervention**				**Pre intervention**				**Post intervention**			
**Group**		**EDI—body dissatisfaction**	**EDI—drive for thinness**	**BIAQ**	**BCQ**	**EDI—body dissatisfaction**	**EDI—drive for thinness**	**BIAQ**	**BCQ**
**INT**	N	32	32	32	32	30	30	30	30
	**M** ***(SD)***	38.22 *(9.66)*	30.19 *(9.25)*	1.90 *(0.67)*	1.99 *(0.98)*	37.90 *(11.73)*	28.50 *(9.79)*	1.80 *(0.70)*	1.90 *(1.00)*
	**Min–max**	9.00–52.00	8.00–42.00	0.47–2.95	0.09–3.74	9.00–53.00	10.00–42.00	0.42–3.05	0.13–3.96
**TAU**	**N**	25	25	25	25	25	25	25	25
	**M** ***(SD)***	41.80 *(9.40)*	33.24 *(8.91)*	2.19 *(0.54)*	2.31 *(0.97)*	41.76 *(9.40)*	33.56 *(9.06)*	2.14 *(0.59)*	2.32 *(1.01)*
	**Min–max**	15.00–53.00	10.00–42.00	1.21–3.00	0.39–3.96	17.00–54.00	9.00–42.00	1.11–3.05	0.26–3.87

Overview of questionnaire scores by group (INT, intervention group; TAU, treatment-as-usual group) and time (pre and post intervention) with mean (M) and *standard deviation (SD)*.

### 3.3 Attentional bias

Pre intervention AB scores confirmed the presence of an AB, as mean AB scores were consistently > 1 (see Table 3).

**Table 3:** Assessing the presence of an AB.

**Group**	**AB view**	**N**	**AB score**
			**M *(SD)***
**AN-INT**	Frontal	23	1.76 *(0.53)*
**AN-TAU**		22	1.89 *(0.58)*
**AN-INT**	Lateral	23	1.85 *(0.70)*
**AN-TAU**		21	2.08 *(0.82)*

Mean (M) and *standard deviation (SD)* of the pre intervention AB scores in frontal and lateral view of the INT and TAU groups (INT, intervention group; TAU, treatment-as-usual group).

Changes in the AB were analyzed using the AB scores in linear mixed models (LMMs) analyses. For the first LMM analysis, group and time were used as fixed factors and the participants' intercept was used as the random factor. The dependent variable were the AB scores. Questionnaire scores (EDI subscales, BIAQ, BCQ) were added as covariates. This was done for both frontal, as well as lateral view. The covariates showed no significant relevance and were consequently removed from the model. This was done for both frontal, as well as lateral view. While no significant effects were found in any of these analyses; there was a trend for time (pre vs. post intervention) with *F*_(1, 41.24)_ = 3.14 and *p* = 0.084 in frontal view (lower scores at post session). See Tables A2, A3, respectively.

Next, another LMM analysis was employed using only INT group data. AB number (pre vs. post session) and session number (1–4) were used as fixed factors. Participants' intercept was used as a random factor again and questionnaire scores were added as covariates. Again, none of the covariates had a significant effect on the outcome; consequently, these were removed. The analysis showed no significant effects for frontal or lateral view (see Tables A4, A5, respectively).

Thereafter, pre intervention AB scores (frontal and lateral view) were correlated with pre intervention questionnaire scores. The correlation scores ranged from *r*_(43)_ = −0.042 to *r*_(43)_ = 0.171 in frontal view and *r*_(43)_ = −0.028 to *r*_(43)_ = 0.154 in lateral view; the significance was always at *p* > 0.05, suggesting that AB scores and AN psychopathology did not correlate significantly (see Table A6 for full results).

### 3.4 Emotions

**Figure 2:**
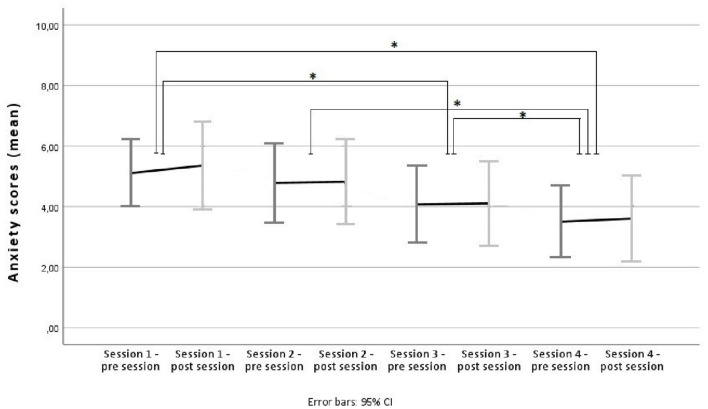
Anxiety scores across sessions. Average anxiety scores pre/post session for sessions one to four, using mean scores and 95% confidence intervals as error bars. Significant changes between overall session scores are marked with an Asterix.

LMM analyses showed that anxiety scores reduced significantly between sessions with *F*_(3, 217.10)_ = 17.17 and *p* < 0.001 (see Table A7). Bonferroni-corrected pairwise comparisons (*post-hoc* tests) revealed significant changes in anxiety scores between sessions one and three, one and four, two and four, and sessions three and four (see Table A8). No changes of anxiety scores within session and no interaction between session number (between-session) and pre/post session (within-session) could be found (see Table A7). See Figure 2 for a visual overview.

**Figure 3:**
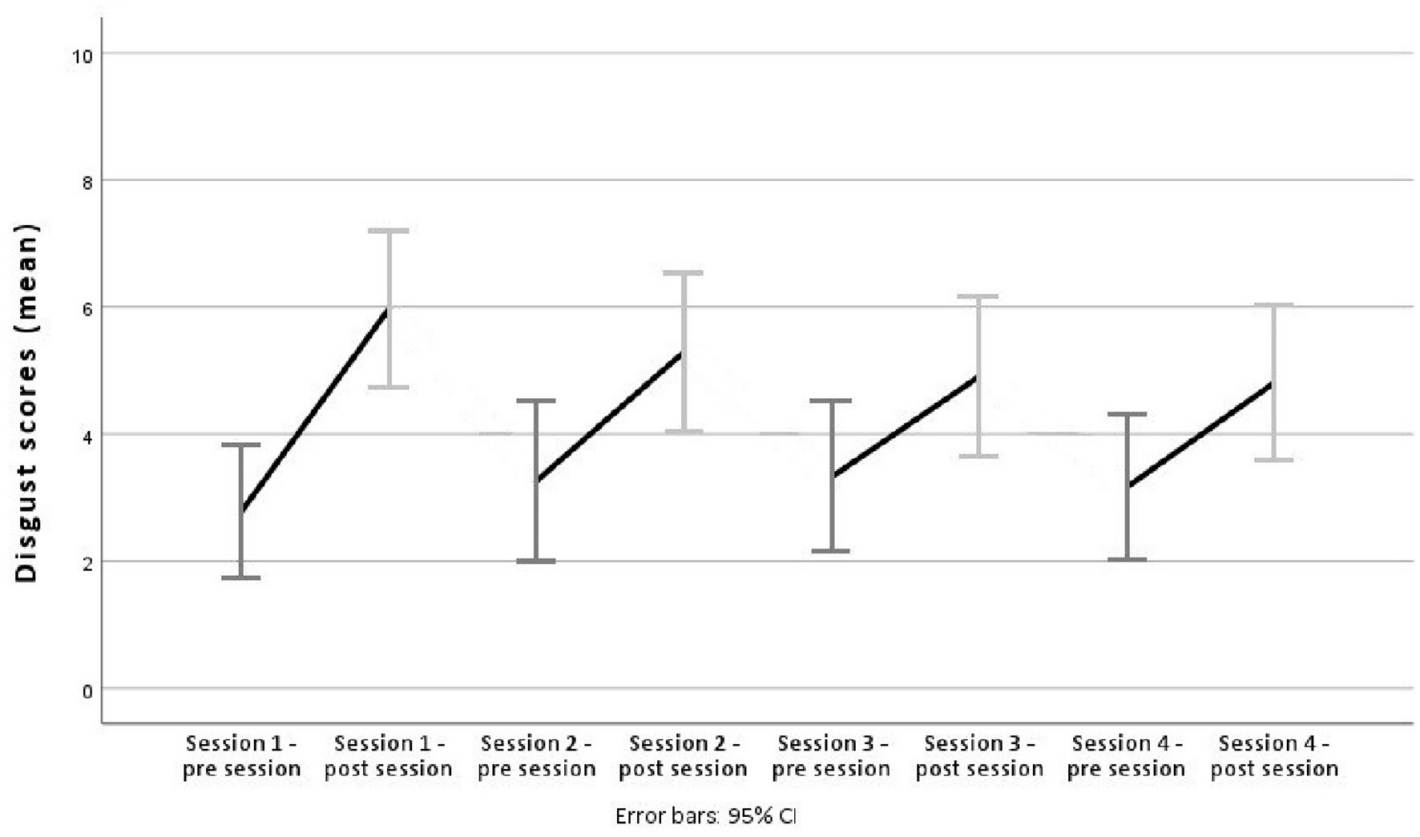
Disgust scores across sessions. Average disgust scores pre/post session for sessions one to four, using mean scores and 95% confidence intervals as error bars.

Results for disgust scores showed a different pattern, as there were no changes between sessions, but a significant increase of disgust within sessions [*F*_(1, 219.97)_ = 115.88 and *p* < 0.001], see Figure 3. Bonferroni-corrected *post-hoc* tests of pre vs. post session scores showed a *MD* = −2.00 (range 2.37–1.64), *SE* = 0.19, *df* = 219.97 and *p* < 0.001. There was also an interaction between session number and pre/post session with *F*_(3, 219.97)_ = 3.42 and *p* = 0.018. *Post-hoc* tests of post session 1 vs. 3 scores showed a *MD* = 1.03 (range 0.05–2.01), *SE* = 0.37, *df* = 219.97 and *p* = 0.034. *Post-hoc* tests of post session 1 vs. 4 scores showed a *MD* = 1.06 (range 0.06–2.06), *SE* = 0.38, *df* = 220.07 and *p* = 0.031. These results indicate that these comparisons in particular seem to be responsible for the aforementioned interaction. See Table A10 for full results of *post-hoc* tests.

## 4 Discussion

The study compared adolescents with AN in an intervention (INT) group to a treatment-as-usual (TAU) group. The INT group received four sessions of computer-based body exposures, while the TAU group did not. As recommended by Tuschen-Caffier et al. (2015), attention was directed to all body parts, so that participants were put off focusing solely on their shape and weight-displaying body parts. We were not able to find any significant improvements in body dissatisfaction or drive for thinness (EDI-2 subscales); body image avoidance (BIAQ); or body checking (BCQ) behaviors in either group (hypothesis 1). There are other studies, in which no changes in ED symptomatology after exposure training (e.g., ABMT) were found (Smeets et al., 2011; Porras-García et al., 2021). For example, Porras-García et al. (2021) also used a subscale of the EDI. It is unclear whether the previously mentioned constructs, which are recorded in the questionnaires, can be changed within a period of 2–3 weeks or whether the used questionnaires are sensitive enough to track small, short-term changes in participants' psychopathology, respectively. Perhaps, using different questionnaires, such as the body image states scale (Cash et al., 2002) which was used by Vocks et al. (2009), would be better to identify such changes.

We were able to replicate findings of (adolescent) patients with AN exhibiting a strong AB toward disliked and weight-displaying body parts (Kerr-Gaffney et al., 2018; Toh et al., 2020; Horndasch et al., 2012). However, upon analyzing our data in terms of a potential reduction of the AB in the INT group, no significant change pre to post intervention, nor an interaction with the TAU group, could be found (hypothesis 2). Nevertheless, a small trend was found for a reduction of the AB in frontal view in the pre to post intervention comparison for both groups. These findings make sense for the INT group, as the body exposures of the intervention are completed in frontal view. However, this trend was also present in the TAU group, which suggests that this slight change might not be due to the body exposures in the intervention, but rather a general treatment effect. This was previously also observed by Kampmann et al. (2018) in a study on social anxiety disorder when comparing participants in an exposure therapy condition to participants in a waiting-list condition.

Analyses of the AB scores from the INT group within and between sessions revealed no significant reduction of the AB (hypothesis 3). We also found no correlation between AN-psychopathology (as measured by the abovementioned questionnaires) and AB scores, i.e., a stronger AB did not correspond to higher AN-psychopathology (hypothesis 4). Ferrer-Garcia et al. (2021) mention that a stronger AB pre exposure therapy is often connected to a lower decrease of ED symptoms during it. Since participants mostly exhibited a strong AB, it may be that we found no changes in the strength of the AB or the questionnaires because of this characteristic. Nevertheless, it should be mentioned that, in some instances, it was found that participants' body dissatisfaction was reduced during body exposure, even though their AB did not change (Vocks et al., 2007; Smeets et al., 2011; Porras-García et al., 2021). Vocks et al. (2018) themselves state that their intervention had the least effect on participants' body perception, while improving cognitive-affective as well as behavioral aspects of EDs in participants. Also, during specific ED therapy, different levels of emotion and attention processing might show a different temporal trajectory with one system changing earlier or later than the other. Attentional biases to social affective pictorial stimuli appear to be more “trait-like” and associated with a lifetime history of AN, whereas emotion regulation difficulties appear to remit during treatment (Harrison et al., 2010). More research is needed to clarify these findings. There was a significant between-session decline of anxiety scores (hypothesis 5), which is in line with, e.g., Melles and Jansen (2023). On the other hand, disgust scores seem to have been activated within each body exposure, as pre to post session analyses consistently showed higher scores at the post session ratings, but which had then decreased again until the next pre session rating (hypothesis 5). Nevertheless, the activation pattern reduced across sessions so that the disgust response was activated less in session four compared to session one, for example. Based on the analyses of the anxiety and disgust scores, it can be said that there was a subjective benefit of the intervention for participants, even though this effect does not seem to have translated to bigger changes in ED pathology or a reduction of the AB. Especially the anxiety reduction should not be disregarded as a small benefit, as this would be a primary outcome for, e.g., anxiety-related exposure therapy (see Meyerbröker and Emmelkamp, 2010; Opriş et al., 2012).

The finding of reduced subjective emotional load but no changes in AB is consistent with reports on a disconnection between different emotion processing levels in AN. A distinct pattern of higher subjective emotion ratings without corresponding physiological responses has been found (Nandrino et al., 2012) e.g., for skin conductance responses, but also for visual stimuli more generally across different processing levels—albeit to a greater extent for food than body stimuli (Burmester et al., 2021), so more research into different processing levels during the specific intervention of body exposure is needed to determine the possible interactions between these. Strengths of our study included the randomized groups, which matched each other in terms of disorders, psychopathology and other demographic features (please see Table 1). Another strength was the standardized procedure of the intervention sessions, which included set time frames and the adherence to a pre-defined protocol according to which the sessions took place. All study attendants used the same pre-recorded audio guidance to facilitate the exposures and the large majority of participants always saw the same study attendant, while a few participants saw a maximum of two study attendants. Unfortunately, we were unable to control for instances in which sessions could not take place or had to be postponed due to illness, participants' distress (e.g., suicidal thoughts) and such. Limitations of our study included small sample size, because of which we were unable to, e.g., analyze subgroups of participants, such as comparing those with typical AN to those with atypical AN, as has been called for, e.g., by Meneguzzo et al. (2023); comparing those with restrictive AN to those with binge/purge-type AN; or analyzing different age groups. Also, participants were not evaluated for alexithymia prior to participation, the reduced ability to describe emotional states often found in EDs might have influenced the results. Participants were neither asked about their sexual orientation nor explicitly about their gender; however, none of the participants showed gender incongruence in the clinical setting in the course of a detailed child and adolescent psychiatric diagnosis. Unfortunately, some of the eyetracking data was of poor quality. Furthermore, the INT group did not record a viewing pattern at the post session, which would have made their data more comparable to that of the TAU group. Moreover, it would have been useful to provide participants with more than four intervention session, which was beyond the scope of our intervention. Subsequently evaluating our study, we agree with Vocks et al. (2007) that more exposures would likely be more effective. In comparison with Biney et al. (2021) whose PBI study comprised of fourteen session, targeting cognitive, affective, and behavioral aspects, including six sessions of mirror exposures, our study seems fairly short. The same applies in regard to the teenBodyWise study by Rosewall et al. (2020), which consisted of eight sessions focused on psycho-educative content about body image. Ascione et al. (2023), who combined an attentional bias modification treatment (ABMT) with an eyetracking paradigm in a virtual reality (VR) setting, showed that it is possible to change the AB in adolescents with AN, even in a single session setting. They highlight the usefulness of ABMT in this context, which seems to be a promising instrument for future treatment options, especially when combined with VR (see also Porras-García et al., 2021).

Finally, we can say that our intervention was not able to change participants' ED symptomatology such as body dissatisfaction, drive for thinness, body image avoidance or body checking behaviors. We were able to confirm the existence of an AB toward disliked and weight-related areas of the body in adolescents with AN; however, our intervention did not significantly reduce this AB. For future research, it might be useful to carry out more body exposure sessions and to incorporate ABMT into an intervention paradigm.

## Data Availability

The raw data supporting the conclusions of this article will be made available by the authors, without undue reservation.
