# Supplementary material for: Evaluating a computer-based body exposure paradigm for the treatment of body image disturbance in adolescent Anorexia Nervosa: effects on the attentional bias and emotions
Source: Front Psychol. 2024 Dec 16;15:1483623. doi: 10.3389/fpsyg.2024.1483623 (PMC11683846; doi:10.3389/fpsyg.2024.1483623)
Supplement: Supplementary file 1 [file Data_Sheet_1.PDF]

Supplementary Materials

Full results of statistical analyses

Table A1

Psychopathology

		F	df	p	η^2
**EDI-2 (subscales)**					
**- body dissatisfaction**	group	1.78	1, 53	.188	.032
	time	0.23	1, 53	.636	.004
	group x time	0.17	1, 53	.679	.003
**- drive for thinness**	group	2.80	1, 53	.100	.050
	time	0.83	1, 53	.366	.015
	group x time	1.73	1, 53	.195	.032
**BIAQ (total score)**	group	3.62	1, 53	.063	.064
	time	3.17	1, 53	.081	.056
	group x time	0.35	1, 53	.558	.007
**BCQ (total score)**	group	1.69	1, 53	.199	.031
	time	1.40	1, 53	.242	.026
	group x time	1.96	1, 53	.168	.036

Notes. Changes in psychopathology according to questionnaire scores (EDI-subscales, BIAQ, BCQ). Results of a 2x2 mixed ANOVA with between-factor: group (INT/ TAU) and within-factor: time (pre/ post intervention). $n_{INT} = 30$; $n_{TAU} = 25$.

Table A2

AB analysis: frontal view (group comparison)

**Fixed effects**	**F**	**df**	**p**
Group	0.83	1, 42.66	.368
Time	3.14	1, 41.24	.084
Interaction: group * time	0.05	1, 41.24	.819
**Random effects**	**Estimate**	**SE**	**p**
Residual	0.20	0.04	<.001
Intercept (ID)	0.19	0.07	.004
	**Score**		
**ICC**	.515		

Notes. Results of linear mixed models analyses of frontal view eyetracking scores with fixed factors group: INT vs. TAU and time: pre vs. post and participants' intercept as a random factor.

Table A3

AB analysis: lateral view (group comparison)

**Fixed effects**	**F**	**df**	**p**
Group	1.10	1, 40.80	.300
Time	0.06	1, 39.03	.810
Interaction: group * time	0.28	1, 39.03	.597

Random effects	Estimate	SE	p
Residual	0.43	0.10	<.001
Intercept (ID)	0.33	0.13	.013
	**Score**		
**ICC**	.395		

Notes. Results of linear mixed models analyses of lateral view eyetracking scores with fixed factors group: INT vs. TAU and time: pre vs. post intervention and participants' intercept as a random factor.

Table A4

AB analysis: frontal view (across sessions; INT group only)

Fixed effects	F	df	p
AB number (pre/ post)	2.03	1, 145.53	.157
Session number (1-4)	1.30	3, 145.61	.276
Interaction: AB number * session number	0.62	3, 145.38	.606
**Random effects**	**Estimate**	**SE**	**p**
Residual	0.25	0.03	<.001
Intercept (ID)	0.27	0.09	.003
	**Score**		
**ICC**	.491		

Notes. Results of LMM analysis of AB scores in the INT-group in frontal view with fixed factors: AB number and session number and participants' intercept as a random factor.

Table A5

AB analysis: lateral view (across sessions; INT group only)

Fixed effects	F	df	p
AB number (pre/ post)	0.00	1, 143.56	.950
Session number (1-4)	0.73	3, 144.05	.537
Interaction: AB number * session number	1.71	3, 143.74	.168
**Random effects**	**Estimate**	**SE**	**p**
Residual	0.39	0.05	<.001
Intercept (ID)	0.26	0.09	.005
	**Score**		
**ICC**	.439		

Notes. Results of AB scores in the INT-group in lateral view with fixed factors: AB number and session number and participants' intercept as the random factor.

Table A6

Correlations of AB and questionnaire scores

Fixed effects	F	df	p
Session number (1-4)	17.17	3, 217.10	<.001
Pre/ post session	0.00	1, 217.03	.978
Interaction: session number * pre/ post session	0.03	3, 217.03	.993
**Random effects**	**Estimate**	**SE**	**p**
Residual	1.80	0.17	<.001
Intercept (ID)	9.55	2.45	<.001
	**Score**		
**ICC**	.841		

		EDI – body dissatisfaction	EDI – drive for thinness	BIAQ	BCQ
**AB frontal (pre intervention)**	r	.090	.074	-.042	.171
	p	.279	.315	.393	.131
	n	45	45	45	45
**AB lateral (pre intervention)**	r	.154	.150	.060	.028
	p	.159	.165	.350	.427
	n	44	44	44	44

Notes. Correlations between AB scores and AN-psychopathology (according to questionnaire scores), using Pearson's r.

Table A7

Anxiety scores: LMM results (INT-group only)

Notes. LMM analysis of anxiety scores across sessions and in pre vs. post session comparison with n = 33.

Table A8

Anxiety scores: pairwise comparisons by session number

Session	MD	SE	df	p	Range of 95% confidence interval
**1 vs. 2**	0.55	0.24	217.03	.125	-0.08 – 1.18
**1 vs. 3**	0.99	0.24	217.03	<.001	0.36 – 1.61
**1 vs. 4**	1.66	0.24	217.16	<.001	1.02 – 2.30
**2 vs. 3**	0.44	0.24	217.03	.384	-0.19 – 1.07
**2 vs. 4**	1.11	0.24	217.16	<.001	0.47 – 1.75
**3 vs. 4**	0.67	0.24	217.16	.033	0.04 – 1.31

Notes. Bonferroni-corrected post-hoc tests for session number comparisons using mean differences (MD).

Table A9

Disgust scores: LMM results (INT-group only)

**Fixed effects**	**F**	**df**	**p**
Session number (1-4)	0.83	3, 220.06	.477
Pre/ post session	115.88	1, 219.97	<.001
Interaction: session number * pre/ post session	3.42	3, 219.97	.018
**Random effects**	**Estimate**	**SE**	**p**
Residual	2.25	0.21	<.001
Intercept (ID)	8.83	2.28	<.001
	**Score**		
ICC	.797		

Notes. LMM analysis of disgust scores across sessions and in pre vs. post session comparison with n = 33.

Table A10

Disgust scores: pairwise comparisons by session number and pre/ post session

	**Session**	**MD**	**SE**	**df**	**p**	**Range of 95% confidence interval**
**Pre session**	**1 vs. 2**	-0.46	0.37	219.97	1.00	-1.44 – 0.53
	**1 vs. 3**	-0.49	0.37	219.97	1.00	-1.47 – 0.50
	**1 vs. 4**	-0.29	0.38	220.07	1.00	-1.29 – 071
	**2 vs. 3**	-0.03	0.37	219.97	1.00	-1.01 – 0.95
	**2 vs. 4**	0.16	0.38	220.07	1.00	-0.84 – 1.16
	**3 vs. 4**	0.19	0.38	220.07	1.00	-0.81 – 1.19
**Post session**	**1 vs. 2**	-0.67	0.37	219.97	.433	-0.32 – 1.65
	**1 vs. 3**	1.03	0.37	219.97	.034	0.05 – 2.01
	**1 vs. 4**	1.06	0.38	220.07	.031	0.06 – 2.06
	**2 vs. 3**	0.36	0.37	219.97	1.00	-0.62 – 1.35
	**2 vs. 4**	0.40	0.38	220.07	1.00	-0.61 – 1.40
	**3 vs. 4**	0.03	0.38	220.07	1.00	-0.97 – 1.03

Notes. Bonferroni-corrected post-hoc tests for the interaction between session number and pre vs. post session comparisons, using mean differences (MD).
